# Modern Sociology

**Published:** 1901-09-14

**Authors:** 


### MODERN SOCIOLOGY.

### THE CASE OF THE SHOP ASSISTANT.

BY A CORRESPONDENT.

### II.—MEALS.

THERE are three Shops Bills before the country—Lord Avebury's Early Closing Bill, on permissive lines; Mr. Provand's amendment of the existing Shop Hours' Act, making it apply to all women assistants and putting the weekly limit at 68 hours; and Sir Charles Dilke's Bill to amend the law relating to shops. This is of wider scope than either, and besides making early closing compulsory, includes provision for sanitation and ventilation, and inspection by H.M. factory inspectors; prohibits Sunday trading, and the employment of children, and regulates overtime and meal-hours. This last clause is a very important one: "a person employed in or about a shop shall be allowed an interval of not less than one hour between noon and two o'clock in the afternoon for dinner, and an interval of not less than half an hour between four and seven o'clock in the afternoon for tea." How necessary such a clause is may be readily seen.

Gastric disease is painfully common, with both men and women shop-assistants. We have gone carefully through some hundreds of medical certificates which prove this, and Dr. Norman Kerr speaks of "the dyspeptic misery and disease induced by the necessary bolting of food through the far too short period allowed for meals." "Shop assistants," said the Rev. J. W. Horsley before the Lords' Select Committee, "are a poor, anæmic, spindle-shanked flat-chested class."

"The doctor says it's the meals," said a girl whom we questioned. "I've had indigestion ever since I went there, two years and a half ago." At her shop 20 minutes is allowed for breakfast, 25 minutes for dinner, and 5 for washing afterwards, a quarter of an hour for tea, and 10 minutes for supper. The assistants are often called down to serve in the shop, especially during the season and sale-time. "It is a trouble," she says, "to get up at all, we are so busy. I have often not been up to tea or supper first day of sale."

"It must be remembered," says Miss Clara Collet, in the Report to the Royal Commission on Labour (1892), "that in the case of shop assistants living on the premises there is no solid meal in the evening to compensate for a light lunch in the middle of the day, and that those who attempt to eat a good dinner in the very short time allowed for it are liable to indigestion." She adds, "In shops where late hours are the custom on Saturdays, but little time is allowed for supper, and in some cases the girls do not sit down to supper until nearly midnight. . . . They acquire a habit of 'bolting' their food in a remarkably short time; indigestion and anæmia are very common among the girls, and their hasty meals and short time for rest may fairly be assigned as the cause of their illness, and their craving for unwholesome food." We have been told that the habit of sending out for "pick-me-ups" is increasing, especially among women and girls.

A young woman who is constantly under the doctor's care for anæmia and indigestion tells us that if only she might have a little time for rest after her lunch, it would make all the difference in the world. She is forewoman in the workroom, and showwoman as well; she is allowed three quarters of an hour for dinner, and as this is not long enough to go out and get a meal, owing to the necessity of always appearing neat and tidy—hair in curl, indoor shoes, long skirts, &c.—she eats her lunch in an underground room, with no ventilation except what comes over a dust-heap infested with mice. Mice run all over the walls, and get into the pockets of the girls' coats hanging on the pegs. Nobody would believe the smells and stuffiness, she says, where the gas burns all day and no fresh air comes. Girls are always leaving on account of bad ventilation and discomfort.

An hour for dinner, and, as in one case of which we have heard, leave to go for a run in the fresh air before returning to work, would lessen the woes of the shop assistant to a large extent. There remains, however, the frequent complaint of bad cooking and bad management—a matter with which no law can deal. A case came under our notice recently. A young woman suffering from gastric ulcers petitioned for a milk diet, and was told she must eat the ordinary fare or go without. She was then living on Benger's food, which, as she was employed in the refreshment department, she made for herself. "If I were in the drapery," she said, "I would get out of it to-morrow." In a very large West End establishment two of the girls said they never went to supper at all; it was not tempting enough. (The usual fare is bread and butter, cheese, ale or water.) They make toast by the kitchen fire, and they spend about £4 4s. a year on extra food, not including luxuries. Another girl spends 3s. a week on extra food, bacon for breakfast, milk and biscuits during the morning, &c., because the fare provided is not varied enough; and a case has been known where one-third of the salary went in food, besides supplies from a home in the country. In a West End shop the assistants are told that their keep is reckoned at £40 a year. It is demonstrable that an average of ten shillings a head per week is sufficient to provide plenty of good and wholesome food; so the sequel to this ought to be impossible:—"The young ladies often come back after dinner and say they haven't been able to eat anything." In one shop at a seaside town the bread and butter for the day was cut before breakfast, with a most depressing effect on those who had to eat it. "I don't grudge a penny for buns," the assistants say; but a clearer idea of what is due to them, when an employer undertakes to provide them with food, would show that it ought not to be necessary that any part of the salary should be spent on it.

Where the assistants go to dinner in batches, it ought to be possible to open the windows and thoroughly air the dining-room between the relays. This is successfully done at the G.P.O., where the women assistants have their meals in companies. It would be a splendid object-lesson if some such plan of co-operative housekeeping could be put in motion, as exists at the G.P.O. A certain sum is granted; a committee of the clerks caters, engages servants, and regulates the price of the menu. Here is a specimen of the day's fare:—

*MENU.*

*May* 13th, 1901.

*Dinners.*

Stewed Steak. Roast Mutton.

Greens. Potatoes.

Queen's Pudding Apple Tart.

*Luncheons.*

Vegetable Soup (Stock) 1½d. and 2½d.

Rissoles 3½d. Bacon and Tomatoes 3½d.

Cheese Cutlets 3d. Curried Steak 4d.

Ham or Tongue 3d. Boiled Egg 1½d., Poached 2½d.

Half-pay Pudding and Sauce. Milk Pudding.

Stewed Fruit.

*Teas.*

Toast.

Meat and one vegetable cost 6½d.; if with pudding, 7½d.; an additional vegetable, 1d. extra. There are "treats" as funds allow.

This is in striking contrast to the "eternal beef and mutton" of the shop assistant. There is variety and cheapness in the food, the heads of the department are saved all trouble, and the girls have the advantage of the training in domestic economy which they get by serving on the committee.

